# The Effect of Spinal versus General Anesthesia on Quality of Life in Women Undergoing Cesarean Delivery on Maternal Request

**DOI:** 10.7759/cureus.3715

**Published:** 2018-12-11

**Authors:** Sina Ghaffari, Laleh Dehghanpisheh, Fahimeh Tavakkoli, Hilda Mahmoudi

**Affiliations:** 1 Anesthesiology, University of Miami, Miami, USA; 2 Anesthesiology, Shiraz University of Medical Sciences, Shiraz, IRN; 3 Epidemiology and Public Health, Nova Southeastern University School of Osteopathic Medicine, Miami, USA

**Keywords:** health related quality of life, general anesthesia, spinal anesthesia, cesarean delivery on maternal request

## Abstract

Introduction

The proportion of women electing for cesarean delivery has increased in both developed and developing countries. Cesarean delivery on maternal request (CDMR) refers to a primary cesarean delivery performed because the mother requests this method of delivery in the absence of standard medical/obstetrical indications.

Several studies compared anesthesia modalities in cesarean section regarding clinical outcomes such as maternal mortality, post-operative pain and bleeding, but only a few compared health-related quality of life (HRQoL) of women undergoing general anesthesia versus spinal anesthesia. The aim of this study was to determine whether pregnant women who undergo general anesthesia (GA) for cesarean delivery compared with spinal anesthesia (SA) differ regarding their perceived HRQoL.

Methodology

We enrolled 160 pregnant women with American Society of Anesthesiologists (ASA) class II, scheduled for CDMR with GA or SA. Anesthesia modality was based on patient’s preference. Participants assessed their state of health with the EuroQoL-5 Dimensions-3 Levels (EQ-5D-3L) self-administered questionnaire at four time points: six hours before cesarean delivery, 24 hours after cesarean delivery, one week and one month after cesarean delivery. Patients also rated their health on the EQ visual analog scale (EQ-VAS) from 100 mm “best imaginable health state” to 0 mm “worst imaginable health state”.

Results

More women who underwent spinal anesthesia reported “no problem” with regards to “mobility’ (64% vs. 30%, p = 0.00), “usual activities” (90% vs. 38%, p = 0.00), and “pain/discomfort” (20% vs. 5%, p = 0.007). Repeated measurement analysis showed that the two groups started off with the same EQ-VAS score, however, both decreased over time with different slope resulting in different scores at 24 hours after CS. Then the scores increased in both groups over time and ended up being rather close at one month after CS.

Discussion

Unless there is a contraindication, neuraxial anesthesia is the anesthetic technique of choice for cesarean delivery in all parturient in general. This concept is based on more mortality and morbidity that have been seen with general anesthesia in this particular population. Our study demonstrated significant advantages of spinal anesthesia compared to general anesthesia in cesarean section regarding postoperatively perceived HRQoL. We showed that more pregnant women who chose spinal anesthesia as their anesthesia modality reported “no problem” with respect to “mobility” and “Self-care” 24 hours after cesarean section. On the top of that, more women in this group had “no problem” in their “usual activities” at one week and one month after cesarean delivery time points. Moreover, EQ-5D general health score was higher 24 hours after cesarean delivery with regional anesthesia comparing to general anesthesia.

Conclusion

We determined that compared to general anesthesia, spinal anesthesia is the technique of choice for cesarean section because not only it avoids a general anesthetic and the risk of failed intubation, but also because it provides effective pain control, mobility and fast return back to daily activities for new mothers and increase their quality of life.

## Introduction

The proportion of women giving birth by cesarean delivery has increased in both developed and developing countries [1]. One frequently proposed explanation is cesarean delivery on maternal request (CDMR). CDMR refers to a primary cesarean delivery performed because the mother requests this method of delivery in the absence of standard medical/obstetrical indications. The prevalence rate of CDMR in all cesarean deliveries is 1-18% globally and less than 3% in the United States [2, 3].

For CDMR, both general and neuraxial are two anesthesia modalities, which have shown equivocal findings with respect to 1 and 5 minutes Apgar scores, umbilical artery pH values and total time in operating room [4]. Although anesthesia guidelines recommend regional anesthesia for cesarean delivery because of the higher risk of failed intubation, aspiration, intraoperative blood loss and awareness with general anesthesia [4, 5], it is still high rate of using general anesthesia on maternal request for this procedure in both developed and developing countries. In England and Wales, 20% of cesarean deliveries were performed with general anesthesia because of maternal refusal of regional techniques [6]. In the United States, the use of general anesthesia for elective cesarean delivery was reported at the level of 5% of cases. The use of general anesthesia for elective cesarean delivery has been reported to about 15% in Great Britain, 4% in Belgium, 30% in Spain, 34% in Italy, 10% in Germany, and 44% in Czech Republic [7].

Since health care is becoming more and more patient centered, patient-reported outcomes such as Health Related Quality of Life (HRQoL) is becoming increasingly important especially in the area of pregnancy and childbirth [8]. Several studies have compared anesthesia modalities in cesarean delivery regarding clinical outcomes in terms of maternal mortality, post-operative pain and bleeding [9-11], and some other studies have compared the quality of life after cesarean with vaginal delivery [12-14]. However, none of them have compared HRQoL among women undergoing general anesthesia versus spinal anesthesia in cesarean delivery.

The aim of this study was to determine whether pregnant women who undergo general anesthesia (GA) for cesarean delivery compared with spinal anesthesia (SA) differ regarding their perceived HRQoL, which can be explained to pregnant mothers by obstetricians and anesthesiologists in their preoperative visit.

## Materials and methods

This observational cohort study was conducted in a tertiary university affiliated hospital. The study was reviewed and approved by the institutional review board (IRB) and Ethics Committees of Shiraz University of Medical Sciences. We enrolled 160 pregnant women with American Society of Anesthesiologists (ASA) class II status, scheduled for CDMR with GA or SA. Exclusion criteria were refusal to give informed consent or contraindications for neuraxial anesthesia (Intrathecal bupivacaine and meperidine).

We recruited 80 eligible patients in each group. Before enrollment, informed consent was obtained from each woman by the anesthesiology resident or attending, that this person did not have a role in the group assignment. Anesthesia modality was based on patient’s preference, after benefits and hazards of each anesthesia technique were discussed to them. Because of the lower rate of general anesthesia, recruitment in this group took 10 months. Both modes of anesthesia (GA and SA) were standardized and administered in conventional ways. Induction of anesthesia was done by propofol and succinylcholine and 0.05 mg/kg of morphine was given intravenous, 15 minutes to the end of the operation. Spinal anesthesia was given by intrathecal administration of 8 mg bupivacaine 0.5% and 20 microgram of fentanyl. Post-operative analgesia was provided by patient-controlled analgesia in both groups with bolus doses of 1 mg morphine per 15 minutes lock time. Surgeries were performed using the Pfannenstiel incision. An anesthesiology resident obtained demographic information and past obstetric history.

Participants assessed their state of health with the EuroQoL-5 Dimensions-3 Levels (EQ-5D-3L) self-administered questionnaire at four time points: six hours before cesarean delivery, 24 hours after cesarean delivery, one week and one month after delivery.

Instructions for the respondent were included in the questionnaire. A trained nurse handed out the questionnaire and provided more instructions, as needed. A trained nurse filled out the questionnaire through a phone call interview at one week and one-month follow-up.

EQ-5D-3L includes the five dimensions mobility, self-care, usual activities, pain/discomfort, and anxiety/depression rated as “no problems”, “some problems”, or “extreme problems” [15]. Patients also rated their health on the EQ visual analog scale (EQ-VAS) from 100 mm “best imaginable health state” to 0 mm “worst imaginable health state”.

The results are presented as health profile by constructing a table with the frequency of reported problem for each level, for each dimension in each group. We used the Farsi language version of the questionnaire, which is officially approved by EuroQol Group’s Translation Committee.

Descriptive statistics were used to describe the basic characteristics of participants. Data were reported as mean (SD) for continuous variables. Nominal data are presented as numbers and percentages. Chi square test and Fisher exact test were used to analyze categorical data. Continuous data were analyzed by means of Student t-test. Repeated measurement ANOVA was used to evaluate EQ-VAS scores time trend in the two groups. Statistical significance was reported at p < 0.05. All analyses were conducted with the use of Stata software, version 12 (StataCorp, College Station, TX).

## Results

In this study we enrolled 160 pregnant women, eligible for CDMR who chose spinal anesthesia (80 women) or general anesthesia (80 women) as their anesthesia modality of choice. The mean age of women was 29.5 (5.5) with a range of 18 to 42 years old.

There was no statistically significant difference regarding age groups, education level, number of abortions, and number of previous general anesthesia. In the SA group, 30 (37%) of women had the experience of spinal anesthesia before, while this number was 11 (14%) for GA group (p = 0.000). More information is depicted in Table 1.

**Table 1:** Demographic and clinical characteristics of women who underwent spinal anesthesia versus general anesthesia.

		Spinal anesthesia N (%)	General anesthesia N (%)	P value
Age	≤25 y	19 (24)	21 (26)	0.86
	25-35 y	49 (61)	49 (61)	
	≥35 y	12 (15)	10 (12)	
Education	8^th^ grade or less	36 (45)	24 (30)	0.12
	High School	25 (31)	35 (44)	
	University	19 (24)	21 (26)	
Number of Children	0	9 (11)	20 (25)	0.013*
	1	45 (56)	28 (35)	
	≥2	26 (33)	32 (40)	
Abortion	0	59	56	0.96
	1	13	15	
	2	6	7	
	≥3	2	2	
Previous spinal anesthesia	Yes	30 (37)	11 (14)	P = 0.00*
	No	50 (63)	69 (86)	
Previous general anesthesia	Yes	45 (56)	45 (56)	P = 1.00
	No	35 (44)	35 (44)	

Because the reported level 3 problems were low, as suggested by the questionnaire guideline, we dichotomized the EQ-5D levels into “no problem” (level 1) and “problems” (levels 2 or 3).

The EQ-5D dimensions were not statistically different before the cesarean delivery between the two groups.

Regarding mobility in the first 24 hours after cesarean delivery (CD), more women in SA group reported no problems compared to women in the GA group (64% vs. 30% women, P = 0.00). There was no statistical difference in mobility at one week or one month after cesarean delivery. Similarly, the self-care dimension was only different at 24 hours after CS (74% women in SA group reported no problems vs. 48% in the GA group, p = 0.001).

Regarding “usual activities”, more women in SA group reported no problems compared to women in the GA group at one week (90% vs. 38%, p = 0.00) and one month (99% vs. 80%, p = 0.00) after cesarean delivery.

More women who underwent spinal anesthesia reported no pain/discomfort at 24 hours and at one month after CS compared to the GA group, 20% vs. 5% (p = 0.007) and 59% vs. 36% (p = 0.007), respectively.

There was no difference in anxiety/depression dimension between the two groups at all time points. More data are shown in Table 2.

**Table 2:** Frequency (percentage) of reported problems by dimension and anesthesia modality group before and after cesarean section (CS). SAG: Spinal anesthesia group; GAG: General anesthesia group.

		Before CS			24 hours after CS			One week after CS			One month after CS		
EQ-5D Dimension		SAG	GAG	P value	SAG	GAG	P value	SAG	GAG	P value	SAG	GAG	P value
Mobility	No Problems	78 (98%)	76 (95%)	0.68	51 (64%)	24 (30%)	0.00*	79 (99%)	74 (93%)	0.11	80 (100%)	77 (96%)	0.24
	Problems	2 (2%)	4 (5%)		29 (36%)	56 (70%)		1 (1%)	6 (7%)		0 (0%)	3 (4%)	
Self-care	No problems	80 (100%)	78 (98%)	0.49	59 (74%)	38 (48%)	001*	80 (100%)	78 (98%)	0.49	80 (100%)	77 (96%)	0.24
	Problems	0 (0%)	2 (2%)		21 (26%)	42 (52%)		0 (0%)	2 (2%)		0 (0%)	3 (4%)	
Usual activities	No problems	79 (99%)	77 (96%)	0.62	13 (16%)	7 (9%)	0.23	72 (90%)	30 (38%)	.00*	79 (99%)	64 (80%)	.000*
	Problems	1 (1%)	3 (4%)		67 (84%)	73 (91%)		8 (10%)	50 (62%)		1 (1%)	16 (20%)	
Pain/Discomfort	No problems	68 (85%)	61 (76%)	0.23	16 (20%)	4 (5%)	.007*	15 (19%)	11 (14%)	0.52	47 (59%)	29 (36%)	.007*
	Problems	12 (15%)	19 (24%)		64 (80%)	76 (95%)		65 (81%)	69 (86%)		33 (41%)	51 (64%)	
Anxiety/Depression	No problems	50 (63%)	45 (56%)	0.52	75 (94%)	73 (91%)	0.76	65 (81%)	54 (68%)	.069	65 (81%)	54 (68%)	0.069
	Problems	30 (37%)	35 (44%)		5 (6%)	7 (9%)		15 (19%)	26 (32%)		15 (19%)	26 (32%)	

**Figure 1:**
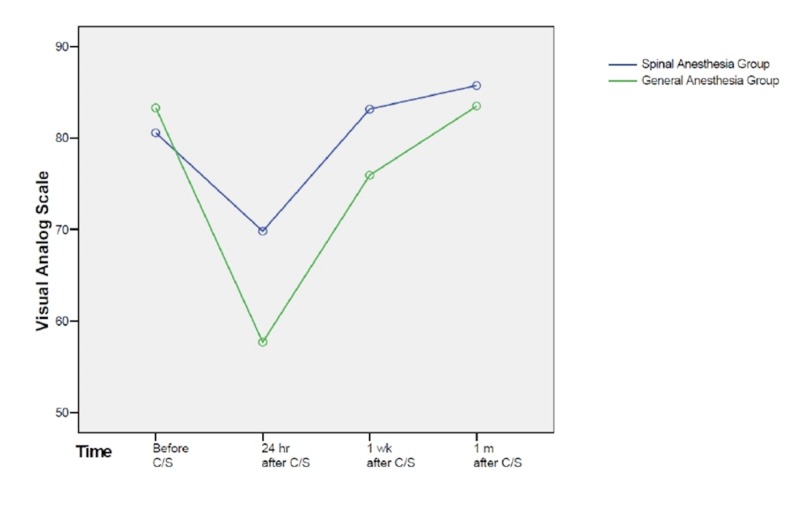
Time trend of EQ-VAS score in spinal anesthesia and general anesthesia groups. EQ-VAS: EQ visual analog scale

In repeated measurement analysis (Figure 1), the between groups test indicated that the effect of “group” was significant (p = 0.006), consequently the graph showed that the lines for the GA group and SA group were rather far apart. The within subject test indicated that there was a significant time effect, in other words, the groups did change over time (p = 0.000), in both groups EQ-VAS score decreased 24 hours after CS and gradually increased over time within one month. Moreover, the effect of interaction between time and group was significant (p = 0.000), suggesting that the effect on groups was not similar over time. The two groups started off with the same EQ-VAS score, however, both decreased over time with different slope resulting in different scores at 24 hours after CS. Then the scores increased in both groups over time and ended up being rather close at one month after CS.

Because the effect of interaction between time and group was significant, we compared the EQ-VAS scores in the two groups at each time point. There was no difference in the mean EQ-VAS score at baseline between the two groups (80.6 ± 11.5 vs. 83.3 ± 17.0 in SA group and GA group, respectively, p = 0.23). At 24 hours after CS, the mean EQ-VAS score was higher in SA group compared to GA group (69.8 (18.8) vs. 57.7 (16.8), p = 0.001). Similarly, EQ-VAS score was higher one week after CS in SA group (83.2 (15.5) vs. 75.9 (18.0), p = 0.007). One month after CS, the mean EQ-VAS scores were 85.75 (17.7) in SA group and 83.5 (20.6) in the GA group, which was not statistically different (p = 0.46). More details are shown in Table 3.

**Table 3:** EQ-VAS score in spinal anesthesia and general anesthesia groups. EQ-VAS: EQ visual analog scale

Time lapse	Spinal anesthesia group Mean (SD)	General anesthesia group Mean (SD)	P-value
Before cesarean section	80.59 ± 11.51	83.31 ± 17.04	0.23
24 hours after cesarean section	69.81 ± 18.85	57.69 ± 16.80	0.000*
One week after cesarean section	83.18 ± 15.58	75.94 ± 18.02	0.007*
One month after cesarean section	85.75 ± 17.72	83.50 ± 20.56	0.46

## Discussion

Our study demonstrated significant advantages of spinal anesthesia compared to general anesthesia for cesarean delivery regarding postoperatively perceived HRQoL. In this study, baseline demographic and obstetric data for the women showed no significant difference between general anesthesia and spinal anesthesia groups. The perceived quality of life level before cesarean delivery measured by EQ-VAS and EQ-5D was also not different between the two groups. The EQ-VAS score decreased after CS in both groups, but spinal anesthesia contributed to higher EQ-VAS score at 24 hours and one week after cesarean delivery.

The American College of Obstetricians and Gynecologists (ACOG) estimates that 2.5% of all births in the United States are cesarean delivery on maternal request [16]. In a 2000 editorial, the former president of the ACOG suggests that perhaps the time had come when the risks, benefits and costs between vaginal and cesarean births are so balanced that the deciding factor could simply be the mother’s preference for how her baby is born. Potential benefits of planned cesarean delivery are known date for delivery, avoidance of post term pregnancy, and neonatal late stillbirth. It also may be associated with decreased risk of pelvic floor injury, early post-partum hemorrhage and unplanned surgeries [2].

Unless there is a contraindication, neuraxial anesthesia is the anesthetic technique of choice for cesarean delivery in most countries [17]. This is based in part on increased mortality and morbidity after general anesthesia in this particular population. Meanwhile, the estimated case fatality rate of general anesthesia during cesarean delivery decreased from 16.8 deaths per million general anesthetics for 1991–1996 to 6.5 deaths per million general anesthetics for 1997–2002. In contrast, the estimated case fatality rate of regional anesthesia during cesarean delivery increased slightly from 2.5 deaths per million regional anesthetics to 3.8 deaths per million regional anesthetics [17]. In addition to emergency situations (35%), maternal refusal (20%) to receive spinal anesthesia is an indication for general anesthesia for cesarean delivery [6].

Previous studies have shown that spinal anesthesia will improve clinical outcomes and decrease the complications in cesarean delivery, but the issue of HRQoL has not previously been evaluated. In 2012, Afolabi and Lesi conducted a systematic review of 20 studies and reviewed 1793 women who underwent cesarean delivery to compare the effect of regional anesthesia versus general anesthesia on the outcomes of cesarean delivery [11]. There is not enough evidence from this review to show that either regional or general anesthesia is superior to the other in terms of major maternal or neonatal outcomes. In that review, only one trial measured satisfaction level using visual analogue score but did not find any differences in satisfaction between regional and general anesthesia. The authors stated that patient satisfaction would need to be evaluated in further researches.

Our results indicate that fewer women who chose spinal anesthesia as their anesthesia modality reported “Pain/Discomfort” at 24 hours and one month after cesarean delivery.

Pain control after CS is important, especially after cesarean delivery because uncontrolled pain not only affects the new mother but also unfavorably influences new born child-care. Neuroaxial anesthesia provides anesthesiologists with an effective and convenient route of opioid administration, and in many countries it is being used as the preferred method of postoperative pain management after cesarean delivery [18]. One of the combinations that are being used for intrathecal injection is bupivacaine and meperidine, which was used in our SA subjects. Meperidine is a synthetic opioid and has been used for analgesia in short day case procedures [19]. The dose varies between 0.5 and 1.0 mg kg^−1^ providing short (4–6 h) duration analgesia. Lemoine et al. [20] showed even low-dose (6 to 7 mg) bupivacaine provides an anesthetic block short enough to allow ambulation within 5 h of cesarean delivery and hospital discharge within 6 h, and lasting long enough to provide analgesia for a surgical procedure of less than 1 h, and for the immediate post-operative phase. Therefore, a successful spinal anesthesia with an appropriate dose of bupivacaine even without an opioid, guarantees immediate post-operative pain relief. In a previous study, spinal anesthesia was shown to be more effective than general anesthesia in terms of pain control during the first two hours post-operatively in transurethral procedures [21]. This is in agreement with our findings in patients with SAG who reported less pain scores immediately after CS. As a further matter, it is not unexpected that women in SAG in our study reported less pain even one month after CS. A retrospective study conducted on 857 subjects who underwent elective cesarean delivery found that the higher pain scores remembered in the immediate postoperative period is an independent risk factor for development of persistent pain after cesarean delivery [22]. Moreover, Eisenach et al. reported that women with severe acute post-partum pain had a 2.5-fold increased risk of persistent pain compared to mild postpartum pain [23].

New mothers also benefit from successful pain management in other ways. It has been shown that successful pain control after cesarean delivery increases the quality of life [24], which is more often accomplished by spinal anesthesia than general anesthesia. A potential explanation for this is that pain relief enables the new mother to be more caring, energetic and active in this period, in which they undertake the role of maternity that consists of many new activities such as nursing and baby care.

In our study, more pregnant women who chose spinal anesthesia as their anesthesia modality reported “no problem” with respect to “mobility” and “Self-care” 24 hours after cesarean delivery. In addition, more women in this group had “no problem” in their “usual activities” at one week and one month after cesarean delivery time points.

It has been shown that compared to general anesthesia, regional anesthesia is associated with significantly less estimated blood loss and lower difference between pre- and post-operative hematocrit [11] which is a major cause of postpartum anemia. Postpartum anemia may cause easy fatigue and loss of energy which interferes with new moms’ activity and mobility and is associated with impaired quality of life [25]. Although we did not collect information about postpartum anemia in our study, this may be a potential reason why more women in general anesthesia group reported problem in terms of usual activity and mobility.

Consistent with our findings, Gursoy et al. showed that neuraxial anesthesia enables patients to return to normal daily activities earlier than general anesthesia. Moreover, the EQ-5D general health score was higher 24 h after cesarean delivery with regional anesthesia compared to general anesthesia [26].

We recognized that there are limitations in our study. Due to the nature of the cohort study design, the pregnant women were not randomized to the intervention and they were assigned to receive general or spinal anesthesia based on their or anesthesia practitioner’s preference, which was unrelated to study. More women in SA group had at least one child compared to the GA group (89% vs. 75%, p = 0.02). Although the correlation between child number and quality of life has not been established [27], having a child may be associated with a higher quality of life in a new mother. This may be because maternal memory and responsiveness increase with each child [28], explaining why women with more maternal experience adapt more naturally to motherhood following the birth of a second child. On the other hand, having existing child/children could also create challenges that would worsen quality of life immediately postpartum.

In our study, more women in the SA group had previous experience of spinal anesthesia compared to GA group (37% vs. 11%), which may be due to high satisfaction level with spinal anesthesia. One study showed that the women who underwent cesarean delivery under spinal anesthesia demonstrated a high rate of patient satisfaction and would choose spinal anesthesia in the future, if required [29].

## Conclusions

In conclusion, we determined that compared to general anesthesia, spinal anesthesia is the technique of choice for cesarean delivery, not only because it avoids the risks of a general anesthetic which includes the risk of failed intubation and its consequences, but also because it provides more effective pain control, early ambulation, hence fast return to daily activities for new mothers thereby increasing their quality of life.
